# Adynamic bone disorder in chronic kidney disease: meta-analysis and narrative review of potential biomarkers as diagnosis and therapeutic targets

**DOI:** 10.1080/0886022X.2025.2530162

**Published:** 2025-07-16

**Authors:** Chia-Ter Chao, Yi-Chou Hou, Min-Tser Liao, Kuo-Wang Tsai, Kuo-Chin Hung, Li-Jane Shih, Kuo-Cheng Lu

**Affiliations:** ^a^ Division of Nephrology, Department of Internal Medicine, Min-Sheng General Hospital, Taoyuan City, Taiwan; ^b^ Nephrology division, Department of Internal Medicine, National Taiwan University Hospital, National Taiwan University College of Medicine, Taipei, Taiwan; ^c^ Graduate Institute of Toxicology, National Taiwan University College of Medicine, Taipei, Taiwan; ^d^ Center of Faculty Development, National Taiwan University College of Medicine, Taipei, Taiwan; ^e^ Division of Nephrology, Department of Internal Medicine, Cardinal-Tien Hospital, School of Medicine, College of Medicine, Fu Jen Catholic University, New Taipei City, Taiwan; ^f^ Department of Pediatrics, Taoyuan Armed Forces General Hospital, Taoyuan City, Taiwan; ^g^ Department of Pediatrics, Tri-Service General Hospital, National Defense Medical Center, Taipei, Taiwan; ^h^ Department of Medical Research, Taipei Tzu Chi Hospital, New Taipei City, Taiwan; ^i^ Department of Pharmacy, Tajen University, Pingtung, Taiwan; ^j^ Department of Medical Laboratory, Taoyuan Armed Forces General Hospital, Longtan, Taoyuan, Taiwan; ^k^ Graduate Institute of Medical Science, National Defense Medical Center, Taipei, Taiwan; ^l^ Division of Nephrology, Department of Medicine, Taipei Tzu Chi Hospital, New Taipei City, Taiwan; ^m^ Division of Nephrology, Department of Medicine, Fu-Jen Catholic University Hospital, School of Medicine, Fu-Jen Catholic University, New Taipei City, Taiwan

**Keywords:** Adynamic bone disorder (ABD), bone-specific alkaline phosphatase (BALP), indoxyl sulfate (is), parathyroid hormone (PTH), protein-bound uremic toxins (PBUTs)

## Abstract

Adynamic bone disorder, common in chronic kidney disease (CKD), results from reduced bone turnover, often due to medications such as calcimimetics or high-dose vitamin D analogs that induce low parathyroid hormone (PTH) levels. Numerous factors contributing to PTH hyporesponsiveness, which also induces low bone turnover, include deficient PTH, uremic toxins like indoxyl sulfate, malnutrition, inflammation, and diabetes. Diagnosis typically involves bone biopsy, although it is inconvenient. Biomarkers like bone-specific alkaline phosphatase (BALP) and intact PTH (iPTH) show promise in distinguishing between low and high bone turnover. Meta-analysis suggests that levels of iPTH below 150 pg/mL or BALP levels below 20 μg/l indicate low bone turnover. Treatments aim to improve bone density without hindering repair, with osteo-anabolic medications being favored for low PTH levels and anti-resorptive agents being cautioned. Romosozumab, while effective, has safety concerns that limit its use. Uremic toxins are reduced by AST-120 treatment, which alleviates PTH hypo-responsiveness and bone toxicity. Adjunctive measures include addressing vitamin D deficiency, and diabetes, and utilizing antioxidant and anti-inflammatory therapies. Overall, BALP and iPTH appear as potential promising biomarkers for diagnosing and monitoring adynamic bone disorder in CKD, effectively guiding therapeutic interventions.

## I. Introduction

Accurate diagnostic tools are crucial in chronic kidney disease (CKD) due to the high prevalence of mineral and bone disorders. Bone turnover markers, which assess osteoblastic and osteoclastic activities, play a key role in predicting fracture risks and guiding the management of CKD-MBD (Mineral and Bone Disorder) [1]. Precise assessment and intervention for adynamic bone disorder (ABD) in CKD demand accuracy, with bone biopsy being the recognized gold standard for obtaining comprehensive insights into bone health [2]. Additionally, bone turnover markers contribute significantly by aiding in fracture risk prediction and the continuous monitoring of bone loss, allowing for personalized treatment strategies [1].

Careful management is essential when aggressively suppressing parathyroid hormone (PTH) levels, as it can benefit some CKD patients but may increase the risk of ABD. The importance of bone markers as treatment targets is underscored in this context [3,4]. These markers are essential for discerning the origins of ABD in advanced CKD, thereby contributing to a nuanced comprehension of the factors that affect bone health [5]. Recognizing their significance, the KDIGO clinical practice guideline emphasizes the role of bone markers in evaluating, preventing, and treating CKD-MBD [6]. Monitoring these markers helps tailor interventions, optimize treatment strategies, and mitigate the increased fracture risk associated with CKD [7].

Diagnosing and treating ABD in CKD relies on pivotal markers like bone-specific alkaline phosphatase (BALP) and intact parathyroid hormone (iPTH), guiding effective interventions [8]. Current research emphasizes emerging biomarkers and tools for diagnosing and managing CKD-MBD, highlighting the ongoing evolution of diagnostic approaches [4,6]. This article analyzes the pathophysiological concept of ABD development, specifically exploring how bone turnover markers, including BALP and iPTH levels, influence the safety and effectiveness of diagnosing and targeting pharmacotherapy in ABD. Furthermore, a meta-analysis utilizing HSROC (Hierarchical Summary Receiver Operating Characteristic) was conducted. This method incorporates hierarchical modeling techniques to address between-study heterogeneity, summarizing the diagnostic accuracy of a biomarker across multiple studies. The aim was to explore the potential positive likelihood ratio (LR+) for low bone turnover disorder of selected biomarkers, which could serve as potential diagnostic and therapeutic targets.

## II. Overview of ABD in CKD

ABD is a significant aspect of CKD-MBD, a complex condition affecting individuals with compromised kidney function [9]. ABD, typified by decreased bone turnover and impaired bone formation, is frequently triggered by the prolonged administration of medications such as calcimimetics and high-dose active vitamin D analogs. This is particularly common in the management of CKD-MBD, especially in cases of secondary hyperparathyroidism (SHPT) characterized by elevated bone turnover [5,10]. This may contribute to the development of hypoparathyroidism. The disruption in bone remodeling increases the susceptibility to fractures, thereby contributing to heightened morbidity and mortality in CKD patients [11,12]. Diagnosis encompasses clinical, biochemical, and radiological assessments, with laboratory tests uncovering reduced PTH levels and decreased bone turnover markers [13]. Confirmation may require bone biopsies for detailed histomorphometry assessment [14]. Managing ABD entails meticulous adjustments to medications impacting mineral and bone metabolism, emphasizing the delicate balance needed to prevent excessive bone resorption and suppressed formation [2]. Presently, a lack of clearly defined diagnostic and treatment targets for bone turnover markers in ABD poses challenges.

Described in the 1980s, ABD has become the predominant bone lesion in the early stages of CKD, surpassing osteitis fibrosa cystica [15,16]. Its multifactorial etiology involves factors such as aging, diabetes mellitus, and relative hypoparathyroidism [8]. ABD is closely linked to calcium overload and the excessive use of anti-parathyroid agents in CKD, potentially leading to increased mortality, fractures, and accelerated vascular calcification [17]. The clinical evaluation for potential ABD usually includes assessing low levels of PTH and alkaline phosphatase (ALP), though bone biopsy continues to be the preferred gold standard [18].

The well-documented multifactorial hypo-responsiveness to PTH is observed in CKD. A certain level of SHPT is advantageous, not only for the positive impact on phosphate levels but also for preserving normal bone formation [19–21]. Low bone turnover results in prolonged secondary mineralization, leading to bone fragility [22]. Higher bone turnover shortens the development time of secondary mineralization, whereas lower bone turnover prolongs its duration [23,24]. The 2017 KDIGO CKD-MBD guidelines advise conducting a bone biopsy before initiating anti-resorptive therapy in CKD G4 to G5D patients with low BMD or fractures. This helps assess whether CKD-MBD, including elevated PTH, contributes to low BMD [25]. There are concerns that bisphosphonates can cause ABD, but studies have not yet clearly linked them to ABD in CKD [26,27]. Bisphosphonates impede bone turnover and reduce the risk of fractures, yet their impact on bone strength is a matter of controversy [5]. Low iPTH levels in CKD patients correlate with increased fracture risk, but it’s unclear if this risk is due to low bone turnover or the underlying disease [5]. In ABD, an imbalance inhibits bone formation *via* low anabolic factors and increased turnover inhibitors [28]. The dual-action treatment of osteoporosis with an anti-sclerostin antibody demonstrates potential in addressing progressive renal osteodystrophy (ROD) in a rat model. This is particularly evident in rats exhibiting low IPTH levels, effectively preventing vascular calcification. Conversely, there were no notable effects observed in animals with elevated PTH levels [29].

## III. Prevalence and histology diagnostic features

The histological bone condition ABD is marked by reduced turnover even with relative normal mineralization. The diagnosis includes a histomorphometric examination of a bone biopsy acquired from the front of the iliac crest [30]. Although the iliac crest biopsy is widely accepted as the gold standard, it recognizes that there is considerable variation in trabecular bone volume among individuals at various locations. It might not reliably predict patient-relevant outcomes such as hip or spine fractures [29]. Despite this, iliac crest biopsy is still valuable for diagnosing mineral metabolism abnormalities, ambiguous mineral metabolism abnormalities, pathologic fractures, or mineral metabolism issues in CKD patients [31]. The prevalence rates of ABD vary due to differing diagnostic criteria, parameters, and cutoff levels, creating inconsistency. The initial definition of ABD was based on the mineral apposition rate, which falls below the lowest value in healthy subjects [32].

Monier-Faugere and Malluche note that ABD is marked by a substantial reduction in bone turnover, including decreased osteoid and bone cells, along with a notable decline in active remodeling sites and tetracycline uptake [33]. Subsequent studies have defined ABD more consistently, frequently relying on a single quantitative parameter that is focused on bone formation. Nevertheless, the differentiation between ABD and other types of ROD became arbitrary [34] with variations from below the median to patients more than one standard deviation below the mean or even beyond the normal range [35,36]. The broad range in defining ABD may impede making clear statements about its epidemiology and its correlation with outcomes. Despite its definition shortcomings, ABD recognized increasingly and emerged as the most prevalent form of renal bone disorder, reaching up to 60% prevalence in Caucasian hemodialysis patients [15,37]. The difficulty in establishing the true prevalence of significantly decreased bone turnover in CKD arises from restricted access to bone biopsy procedures in large, unselected populations.

## IV. Cause of ABD and bone loss in CKD

Low bone turnover in CKD is associated with advanced age, comorbidities, malnutrition, inflammation, and hypoalbuminemia [38]. This bone characteristic is influenced by various factors, including deficient PTH signaling, exposure to aluminum, protein-bound uremic toxins (PBUTs), and diabetes mellitus [17,39–41]. Additionally, an over-suppression of PTH levels, whether through calcimimetics, high doses of active vitamin D analogs, or parathyroidectomy, can result in decreased PTH levels and responsiveness. The following list outlines the primary factors that contribute to this decline in PTH sensitivity.

### IV-1. Protein-bound uremic toxins (PBUTs)

In CKD, the accumulation of protein-bound uremic toxins (PBUTs), particularly indoxyl sulfate (IS), leads to low bone turnover. IS contributes to low bone turnover by inhibiting bone formation through the downregulation of PTH receptors [42] and suppressing WNT signaling in osteoblasts [43]. The ingestion of an intestinal adsorbent (AST-120) decreases uremic toxins, leading to an increase in bone turnover in rats with uremia [44]. PBUTs and PTH impact bone metabolism differently in various stages of CKD. It is widely recognized that during the early stages (2-3) of CKD, Wnt signaling inhibitors from the kidney or bone cells reduce osteoblast viability, leading to impaired bone architecture [40]. We previously also found osteoblast and osteoclast function is further hindered by PBUTs such as IS and p-cresol sulfate (pCS) [41]. In CKD, a disturbance in the gut microbiome results in the buildup of uremic toxins, which is associated with low bone turnover and uremic osteoporosis [45]. Ongoing bone loss is occurring due to the decline in renal function, metabolic acidosis, and hyponatremia [40,46]. The development of the high-turnover bone disorder is a characteristic of advanced stages (4-5) of CKD, resulting from the dysregulation of vitamin D, PTH, minerals, and phosphate. Osteoclast and osteoblast activity is intensified by the overriding of bone formation inhibitors by elevated PTH. However, one cell viability can be restored to a low turnover state by treating secondary hyperparathyroidism [40]. Our previous research demonstrated that the IS/AhR/MAPK signaling pathway hinders osteoblastogenesis. Resveratrol may offer therapeutic potential in restoring osteoblast development decline caused by IS-induced suppression of bone turnover in CKD [47].

### IV-2. Osteoblast hypo-responsive to PTH and attenuate anabolic signaling

In advanced CKD treatment, secondary hyperparathyroidism (SHPT) is commonly managed with potent medications such as calcimimetics or active vitamin D. The administration of exogenous calcium also has proven effective in suppressing PTH. However, the increased occurrence of low bone turnover in these patients raises concerns about the potential for overtreatment of SHPT [39,48]. Furthermore, in addition to the absolute decrease in PTH levels due to treatment, the diminished response of bones to PTH (PTH hypo-responsiveness) also plays a significant role [19,49].

Patients with CKD who display a significant accumulation of PBUTs, like IS, undergo a notable influence on PTH signaling. This influence can potentially lead to the development of adaptive SHPT due to PTH hypo-responsiveness [43]. In osteoblast, PTH activates cAMP through PTH1R, leading to the stabilization of β-catenin and increased bone formation. Wnt ligands, binding to FZD/LRP5/6 receptors, accumulate β-catenin in the nucleus, further promoting bone formation. PTH also hinders sclerostin (SOST), thereby enhancing bone formation. However, in CKD, IS stimulates SOST, inhibiting bone formation. IS also exerts adverse effects on PTH metabolism and signaling. This includes downregulating PTH1R, reducing intracellular cAMP, inducing competitive inhibition between PTH and its fragments on PTH1R, and increasing cellular oxidative stress [43]. Defining optimal IPTH levels, especially at the individual level, is challenging. Developing a widely accessible assay for PTH1 receptor activation is essential. Simultaneously, evaluating specific bone turnover biomarkers or a panel against the gold standard of bone histomorphometry is crucial for advancement [50]. In addition, elevated SOST in early CKD inhibits Wnt/β-catenin, a crucial factor for osteoblast development [51]. In a mouse model of CKD, activin A plays a pivotal role by activating SOST [52]. However, PTH can inhibit sclerostin and affect bone dynamics [53]. Early CKD involves FGF23 inhibiting the WNT/β-catenin pathway through Dickkopf1 activation [54]. Novel anabolic agents demonstrate therapeutic potential by restoring balanced bone turnover, offering promise for improving bone health in individuals affected by CKD [2].

### IV-3. Malnutrition/inflammation attenuates bone turnover

Low bone turnover and an increased risk of fractures are linked to malnutrition and chronic inflammation, which are both components of the malnutrition/inflammation syndrome [55,56]. Low levels of nutritional markers are present in conditions with low PTH states, which contribute to the progression of low bone turnover [57,58]. Inflammatory cytokines have been shown in laboratory studies to contribute to bone resorption and hinder bone formation [59]. Patients undergoing dialysis, who display limited bone turnover, experience diminished *in vitro* growth and elevated interleukin-6 production in osteoblasts [60]. In addition, PTH can be suppressed by inflammatory cytokines, which could result in an indirect decrease in bone turnover [61]. Clinical studies have indicated a positive effect on BMD through the inhibition of inflammatory cytokines [62]. This favorable outcome is likely due to the dual mechanism of limiting bone resorption and enhancing bone formation [63]. Bisphosphonates inhibit pro-inflammatory cytokines, potentially contributing to a positive impact on bone mass [64]. Resveratrol (RSV) mimics the positive effects, promoting osteogenesis in human periodontal ligament stem cells (hPDLSC) despite TNF-α inhibition. It works by activating the ERK1/2 pathway, reducing TNF-α-induced IL-6 and IL-8 secretion, and enhancing osteogenesis [65].

### IV-4. Diabetes mellitus on bone turnover

Diabetes mellitus (DM) worsens CKD-MBD, impacting bone turnover. DM directly reduces bone mass and turnover by affecting osteoblasts and disrupting parathyroid hormone secretion [66]. This dual effect raises the risk of bone mineral disorders and fractures in individuals with diabetes and CKD [67], affecting the bone structure, quality, and density [68]. Insulin signaling in osteoblasts boosts bone turnover by upregulating Runx2 (runt-related transcription factor 2) and downregulating OPG (osteoprotegerin) [69]. The release of undercarboxylated osteocalcin is linked to improved glucose tolerance and accentuated beta-cell function [70]. Observational studies link diabetes mellitus to a higher prevalence in low-turnover groups of renal osteodystrophy [71]. Diabetic osteodystrophy is marked by diminished bone turnover, reduced osteoblast and osteoclast activity, and decreased bone volume [72]. Diabetes-related bone loss is influenced by factors such as hyperglycemia, insulin deficiency, insulin-like growth factor I resistance, advanced glycation end-product accumulation, inflammation, and oxidative stress [73]. In both type 1 and type 2 diabetes, there’s a proposed dissociation in bone turnover, emphasizing a greater inhibition of bone formation than resorption, increasing the risk of bone loss [74–76]. Recognizing these factors highlights the necessity for customized management strategies addressing diabetes-related bone health challenges in CKD [77].

## V. Low bone turnover and clinical outcomes in CKD

Patients with CKD and low bone turnover may have decreased BMD, linked to altered bone turnover favoring resorption, potentially due to chronic inflammation and suppressed WNT/β-catenin signaling [78]. Dual-energy X-ray absorptiometry (DXA) is valuable in predicting fracture risk in moderate to advanced CKD. It assists in identifying fractures and assessing bone health [79]. Biomarkers like lower FGF-23, higher α-Klotho, and lower IPTH are specific indicators of ABD [80], supporting the use of DXA for predicting fracture risk in advanced CKD [25]. In low bone turnover states like ABD, there is an observed reduction in the bone’s ability to buffer calcium flux. This connection links increased calcium exposure to vascular calcification [81]. Studies in CKD patients establish links between low bone turnover or low PTH levels and arterial calcification [82,83]. Reducing dialysate calcium levels in hemodialysis (HD) patients with low bone turnover decelerates the progression of coronary artery calcification and enhances bone turnover [82]. However, the impact of calcium supplementation on negative outcomes in CKD remains uncertain, with conflicting findings in meta-analyses and recent observational studies [84,85].

Prolonged or excessive mineralization, known as ‘over-mineralization,’ from slow bone remodeling, can lead to brittle bones and increased fracture risk [86]. Although inhibiting remodeling may lead to the accumulation of microcracks, its effect on bone strength and fracture rates remains uncertain [87]. The relationship between PTH and bony fracture risk varies, with elevated IPTH linked to increased risk [88,89]. Alkaline phosphatase, especially bone isoforms affecting total serum levels, consistently associates with outcomes in CKD, suggesting a potential link between low PTH, bone fragility, and cardiovascular outcomes [90].

Despite the variability, bone turnover markers like BALP and TRACP-5b may predict cardiovascular morbidity and mortality in CKD [91,92]. Post-parathyroidectomy, low PTH correlates with improved survival and vascular outcomes [93,94]. Vascular calcification regresses after parathyroidectomy, even with calcium loading for the hungry bone syndrome [95]. Bisphosphonates and denosumab, common in osteoporosis treatment, reduce fracture risk in postmenopausal women without reported accelerated vascular calcification [96]. Studies in advanced CKD patients show no vascular safety signals [97,98]. While concerns about the long-term use of bisphosphonates increasing fracture risk persist, reassuring findings suggest the safety and benefits of denosumab for up to 10 years in postmenopausal women [99,100].

## VI. Role of bone turnover biomarkers in advanced CKD

Managing CKD-MBD focuses on bone turnover abnormalities to prevent low bone mass and fractures. Distinct treatments are required for low and high bone turnover [37,79]. In clinical settings, PTH is a frequently used marker. However, KDIGO advises against specific PTH targets due to inconsistent histological reflections of bone architecture and turnover [101]. However, the limited clinical use of turnover markers, affected by kidney clearance, makes establishing concentration targets unreliable [29].

### VI-1. Potential biomarkers identified for ABD diagnosis

Several potential biomarkers have been identified in previous studies for the expected diagnosis of ABD. These biomarkers play a crucial role in assessing bone turnover and mineralization, helping clinicians identify ABD in CKD patients (Table 1).

**Table 1.** The proposed cutoff value of blood iPTH and BALP level for the potential diagnosis of adynamic bone disease (ABD) based on bone biopsy in CKD.

Study	Study Subjects	Bone biopsy	Cutoff value or mean value
P Ureña, et al. **1996 [**128] **GII assay**	42 HD patients 10 normal or LTBD	Yes	iPTH ≦ 150 pg/mL (sensitivity of 70%, specificity of 78%) BALP ≦ 20 U/L (sensitivity of 100%, specificity of 100%)
Couttenye MM, et al. **1996 [**226] **GII assay**	103 HD patients 38 with ABD	Yes	iPTH ≦ 150 pg/mL (sensitivity of 80.6%, specificity of 90.6%) BALP ≦ 27 U/L (sensitivity of 86.4%, specificity of 76.2%)
Giorgio Coen, et al. **1998 [**124] **GII assay**	41 HD patients 9 with LBT	Yes	iPTH < 79.7 pg/mL (sensitivity of 88.9%, specificity of 90.6%) BALP: <12.9 ng/mL (sensitivity of 100%, specificity of 93%)
Giorgio Coen, et al. **2002 [**227] **GII assay**	35 HD patients 9 with LBT	Yes	miPTH: 65.22 ± 55.25 pg/mL mALP: 186.64 ± 186.96 U/L mBALP: 28.22 ± 41.73 U/L
Giorgio Coen, et al. **2002 [**132] **GII assay**	79 CKD patients; 107 HD patients	Yes	miPTH: 113.42 ± 126.61 pg/mL mALP: 72.44 ± 36.28 U/L iPTH: <100 pg/mL ALP: <100 U/L
An R.J Bervoets et al. **2003 [**127] **GII assay**	84 CKD3-5 patients 19 with ABD	Yes	iPTH < 237 pg/mL (sensitivity of 78%, specificity of 53%) BALP ≦ 23 U/L (sensitivity of 83%, specificity of 66%)
Gabriele Lehmann, et al. **2005** [133] **GII & GIII assay**	132 CKD3-5 patients	Yes	CKD 3-4: miPTH: 47.5 ± 39.7 pg/mL CKD 5: miPTH: 53.0 ± 44.7 pg/mL iPTH < 86 pg/mL BALP < 23 U/L (sensitivity: 80%, specificity: 95%)
F.C. Barreto, et al. **2008 [**36] **GII assay**	97 HD patients (58 LBT)	Yes	iPTH < 150 pg/mL (sensitivity of 50%, specificity of 85%)
Carol Moore, et al. **2009 [**125] **GIII assay**	43 HD patients (7 ABD)	Yes	miPTH: 225 ± 111 pg/mL median BALP: 16.2 ± 8.1 U/L
Hartmut H Malluche, et al. **2011 [**15] **GII assay**	630 HD patients (338 LBT)	Yes	In white patients mPTH: 172 ± 12 pg/mL mALP: 120 ± 5.52 U/L
Mathias Haarhaus, et al. **2015 [**109] **GII assay**	40 HD patients 21 LBT	Yes	Presence of B1x median iPTH: 49 pg/mL median BALP: 18.6 U/L
Stuart M Sprague, et al. **2016 [**37] **GII assay**	492 HD patients	Yes	iPTH: 103.8 pg/mL (AUROC: 0.701) iPTH < 2 times ULN (sensitivity of 65.7%, specificity of 65.3%) BALP < 33.1 ng/mL (AUROC: 0.757)
Syazrah Salam, et al. **2018** [134] **GII assay**	69 CKD 4-5 patients 11 LTBD	Yes (43 patients)	iPTH ≦ 183 pg/mL (sensitivity of 70%, specificity of 53.6%) BALP ≦21 ng/mL (sensitivity of 89%, specificity of 77%) ALP ≦88 ng/mL (sensitivity of 91%, specificity of 63%)
Emilia M D Soeiro, et al. **2020 [**228] **GII assay**	42 children on dialysis	Yes	iPTH < 2 times ULN associated with low bone turnover (RR 5.62)
Suthanit Laowalert, et al. **2020 [**229] **GII assay**	22 HD patients 10 ABD	Yes	miPTH: 375.22 ± 140.13 pg/mL mBALP: 28.59 ± 15.24 U/L mALP: 87.90 ± 30.72 U/L

**Abbreviations:** ABD, Adynamic Bone Disease; AUROC, area under the receiver operating characteristic; bALP, bone-specific alkaline phosphatase; HD, hemodialysis; CKD, chronic kidney disease; iPTH, intact parathyroid hormone; LBT, low bone turnover; m, mean; RR, risk ratio; **GII**: Second-Generation iPTH Assay, intact PTH assays; **GIII**: Third-Generation iPTH Assay, bio-intact PTH or whole PTH” assays.

#### Parathyroid hormone (PTH)

Plasma iPTH levels below 50 pg/ml are associated with ABD, while levels above 800 pg/ml are linked to high turnover bone disorder. However, levels between 100 and 500 pg/ml exhibit varying associations with different types of bone lesions [8]. A crucial study on 492 dialysis patients determined intact PTH (iPTH) cutoffs at 108 pg/mL for low and 323 pg/mL for high bone turnover [37]. While iPTH is generally correlated with bone turnover, individual variability hinders its reliability as a sole indicator. The sensitivity of KDIGO cut points (2 to 9 times normal) for bone turnover diagnosis was low, but iPTH concentrations exceeding 9 times normal exhibited 86% specificity for the high turnover disorder [37,80]. Therefore, iPTH is a valuable population-level bone turnover marker but may lack precision for individual patients. Extremely high iPTH indicates high turnover, while consistently low levels below 50 pg/mL suggest low turnover with lower specificity [37]. It is important to recognize that current thresholds for iPTH may have limited predictive value in specific populations. In African American patients, for instance, ABD has been observed at higher iPTH levels than KDIGO-recommended ranges [102,103]. Due to potential differences in vitamin D metabolism and PTH responsiveness, bone biopsies may remain necessary for accurate differentiation between high- and low-turnover bone disorders in this population [104].

#### Alkaline phosphatase (ALP)

Alkaline phosphatase is an enzyme that catalyzes the hydrolysis of phosphomonoesters, including the endogenous substrate inorganic pyrophosphate (PPi) [105]. Humans express four ALP isozymes, and among them, bone-specific ALP (BALP) has four isoforms (B/I, B1x, B1, and B2) [106]. In CKD-MBD, ALP exhibits lower variability than IPTH, making it effective for diagnosing and monitoring bone turnover longitudinally [29,106,107]. Elevated blood ALP levels directly mirror bone turnover, indicating bone metabolism and predicting mortality in CKD patients. Serum ALP emerges as a proposed alternative marker for renal bone disease, offering distinct advantages over PTH, with a direct correlation to bone turnover and predictive potential for outcomes in CKD patients [29,108].

#### Bone-specific alkaline phosphatase (BALP)

Among different bone turnover biomarkers, the serum-specific BALP isoform B1x is associated with low bone turnover. In a clinical study on low bone turnover instances, hemodialysis patients typically exhibit a mean iPTH level of 83 pg/mL and a BALP level of 22.4 U/L. B1x proves to be a valuable diagnostic marker for detecting low bone turnover [109]. In a study with 492 dialysis patients, iPTH and BALP effectively distinguish between low and non-low bone turnover, with optimal cutoffs of <103.8 pg/mL for iPTH and <33.1 U/L for BALP [37]. In a group of long-term hemodialysis patients, a robust correlation was found between an iPTH level below 150 pg/dL and the existence of low-turnover bone disorder [110]. Barreto et al. discovered that among dialysis patients with iPTH levels ranging from 150 to 300 pg/mL, 66% exhibited low-turnover bone disorder, and 25% had high-turnover bone disorder [111]. Nevertheless, iPTH levels might more accurately reflect parathyroid activity rather than the underlying bone remodeling [112,113]. iPTH levels within the KDIGO-recommended range have limited predictive value for bone histology [113]. In low bone turnover patients, the mean iPTH was 53.2 pg/mL, BALP was 15.3 U/L, and TRAP5b was 2.7 U/L. Optimal cutoffs for diagnosis were iPTH < 90.5 pg/mL and BALP < 24.2 μg/L, with combinations of markers slightly improving diagnostic performance [114].

Crucial bone formation biomarkers, ALP and BALP, remain unaffected by the glomerular filtration rate [115]. In patients with CKD and those undergoing dialysis, depending on BALP for individual patient assessment has limitations [89,116]. Serum BALP is proposed as an alternative marker due to diagnostic advantages over iPTH [106,117]. High BALP levels practically rule out the presence of ABD [118]. The combination of biochemical markers, such as iPTH plus osteoprotegerin or iPTH plus BALP, holds promise for differentiation [29,119]. The ratio of PTH(1–84) to PTH(7–84) is another approach for assessing bone health [120].

Our prior findings indicated that iPTH levels below 100-150 pg/mL and BALP in the lower quartile of normal values are indicative of low bone turnover disorder [121]. Additionally, another study demonstrated that low bone turnover can be inferred from a combination of BALP levels below the lower limit of the normal reference range and iPTH levels below two times the upper limit of the normal reference range [122]. An ongoing randomized controlled trial aims to assess whether treatment with recombinant human PTH can enhance bone turnover and bone mineral density (BMD), potentially reducing the risk of fractures in CKD 4-5D patients with ABD. Inclusion criteria comprise a T-score ≤-2 on DXA scan of the total hip, femoral neck, or lumbar spine (L1-4), and patients anticipated to have dynamic bone disorder based on BALP ≤21 µg/l or biopsy-confirmed low bone turnover. Patients with serum 25-hydroxyvitamin D2 and D3 < 50 nmol/l are excluded, but they may be reconsidered after addressing vitamin D deficiency. The primary outcome measure is changes in BALP, along with the number of patients no longer exhibiting dynamic bone lesions (BALP >21 µg/l). The expected study completion date is September 1, 2027 (ClinicalTrials.gov identifier: NCT04522622).

### VI-2. Correlation of ALP with bone turnover and fracture risk

Accurate diagnosis of bone turnover and pathology in CKD relies on bone biopsy, providing insights into mineralization and volume [123]. Coen et al. revealed that BALP levels below 12.9 ng/mL effectively predicted low-turnover bone disorder. Utilizing criteria like iPTH ≥ 300 pg/mL, BALP > 20.9 ng/mL, and calcium > 8.4 mg/dL assisted in excluding cases of ABD among participants with hyperparathyroid bone disease [124]. BALP is regarded as more effective in evaluating bone turnover. Combining these markers is recommended for enhanced assessment, though rare discrepancies may occur due to measurement variability [124–128].

Limited research has investigated the predictive potential of iPTH and BALP for fracture incidence. In a study involving 185,277 hemodialysis patients, a significant association was found between higher total ALP and hip fracture incidence, particularly in individuals with lower iPTH levels. Conversely, in the highest iPTH quartile, serum ALP did not independently predict hip fractures [90]. International guidelines are recommended to include ALP as both a risk marker and treatment target due to substantial evidence supporting its significance in bone-related issues, cardiovascular outcomes, and mortality [5,129,130]. CKD patients often experience bone fragility fractures, and using markers like ALP alongside tools such as FRAX helps predict fractures, providing insights into CKD-related bone complications [107,131]. Although there are controversies, the link between bone turnover markers, including ALP, and an elevated risk of fractures is acknowledged in CKD [107]. Monitoring treatment efficiency using these markers before evaluating BMD changes allows for early assessment of pharmaceutical treatment efficacy and reinforcement of patient compliance.

### VI-3. Methods

**Figure 1.**
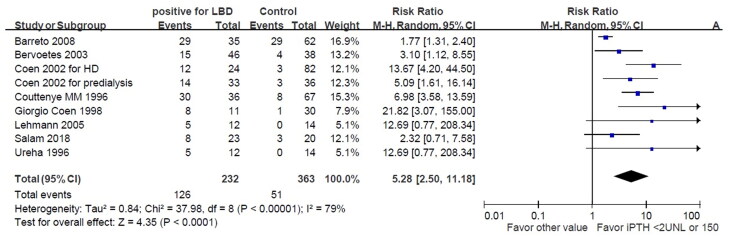
The merged positive likelihood for iPTH < 150 pg/mL or 2 ULN for low bone turnover disorder. The analysis entailed extracting HSROC, sensitivity, and specificity data from relevant studies. We included eight studies to assess the effectiveness of iPTH in diagnosing low bone turnover. The results showed that the combined positive likelihood ratio for iPTH levels below 150 pg/mL or 2ULN in detecting low bone turnover disorder was 5.28 (95%CI: 2.50-11.18).

**Figure 2.**
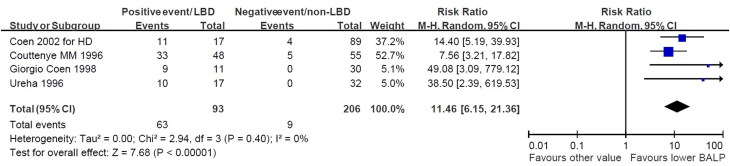
The positive likelihood for BALP is less than 20 μg/l for low bone turnover disorder. The analysis included data extraction on HSROC, sensitivity, and specificity from relevant studies. Specifically, we incorporated four studies to assess the effectiveness of BALP in diagnosing low bone turnover disorder. The results showed that the positive likelihood ratio for low bone turnover with BALP levels below 20 μg/l was 11.46 (95%CI: 6.15-21.36).

We conducted a structured literature search in PubMed and MEDLINE databases up to August 2023 using the following terms and Boolean combinations: *‘Adynamic Bone Disease’ OR ‘low bone turnover’ AND ‘chronic kidney disease’ OR ‘renal osteodystrophy’ AND ‘biomarkers’ OR ‘iPTH’ OR ‘bone-specific alkaline phosphatase’ OR ‘BALP’ AND ‘bone biopsy’*. Only original studies that included bone histomorphometry and reported corresponding serum iPTH and/or BALP levels were considered. The key studies used for generating the forest plots and meta-analyses were selected based on availability of sufficient diagnostic accuracy data (true/false positives and negatives) and inclusion of histological gold-standard references (Table 1). To investigate the potential diagnostic significance of iPTH and BALP levels in ABD based on bone biopsy, we gathered data from 15 studies examining the suggested cutoff values of serum iPTH and BALP levels in CKD (Table 1). We have also refined the inclusion criteria and clarified the data extraction methods for the studies included in the meta-analysis of iPTH and BALP levels, which were used to develop the forest plots (Figures 1 and 2).

### VI-4. The predictive role of the serum iPTH and BALP for low bone-turnover disorder

We performed a meta-analysis to evaluate the predictive accuracy of serum iPTH and BALP in detecting low bone turnover disorder from the studies listed in Table 1 providing true and false positive and negative numbers. After extracting data documenting the HSROC (Hierarchical Summary Receiver Operating Characteristic), sensitivity, and specificity of each parameter, eight studies for iPTH and six studies for BALP were included to assess their efficacy, as depicted in Table 1. The data from Coen et al. [132] were divided into dialysis patients and predialysis patients (labeled as Coen 2002 for HD and Coen for predialysis, respectively). We employed HSROC in meta-analysis to consolidate the diagnostic accuracy of a biomarker across multiple studies, integrating hierarchical modeling techniques to address between-study heterogeneity. We combined sensitivity, specificity, and HSROC data for iPTH levels below 150 pg/mL or less than 2 times the upper limit of normal (2 ULN). The likelihood ratio (LR), a statistical measure utilized to evaluate the diagnostic accuracy of a test, was employed in this study.

As shown in Table 1, each study is annotated to indicate whether a second-generation (GII) or third-generation (GIII) PTH assay was employed. The majority of studies utilized second-generation assays, with only two adopting third-generation methods. Given that ABD is generally associated with low PTH levels, the potential overestimation of iPTH by second-generation assays is unlikely to significantly affect diagnostic accuracy in this context. Furthermore, both I-PTH (second-generation) and BI-PTH (third-generation) assays have been shown to effectively identify patients with low bone turnover and differentiate them from those with high-turnover renal bone disease [133]. We recognize that iPTH thresholds, such as <150 pg/mL, may not be universally applicable across different assay platforms or patient populations. Notably, evidence suggests that African American patients with histologically confirmed adynamic bone disease can exhibit significantly higher iPTH levels when assessed using second-generation assays [15]. Accordingly, the interpretation of PTH levels should be tailored to the individual, taking into account the assay methodology, patient demographics, and relevant clinical context [103].

Figure 1 depicts that the combined positive likelihood ratio of iPTH levels below 150 pg/mL or 2ULN for the low bone turnover disorder was 5.28 (95%CI: 2.50-11.18). To assess the impact of BALP on the accuracy of diagnosing low bone turnover (LBT), we analyzed six studies involving BALP levels below 30 μg/l. The results revealed that the positive likelihood ratio for low bone turnover with BALP levels lower than 30 μg/l was 8.84 (95%CI: 4.47-17.45). Additionally, when we focused on four studies with BALP levels below 20 μg/l, Figure 2 illustrates that the positive likelihood ratio for low bone turnover with BALP levels lower than 20 μg/l was 11.46 (95%CI: 6.15–21.36).

**Figure 3.**
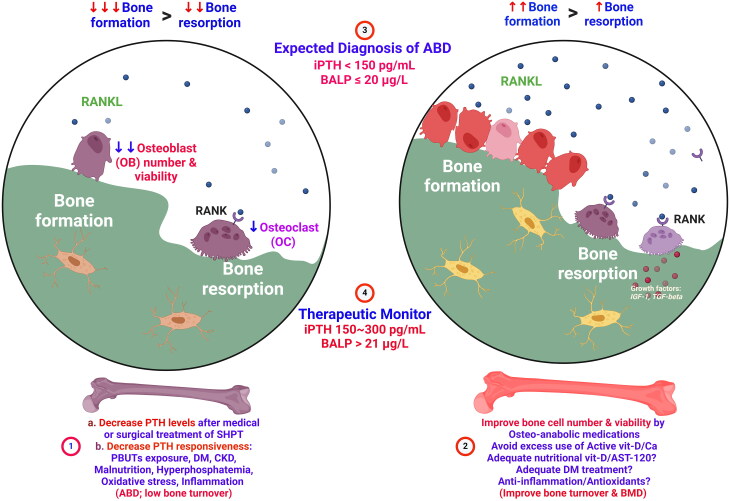
iPTH And BALP levels may be prospective diagnostic and potential therapeutic monitoring biomarkers for Adynamic bone disease (ABD) in CKD. **①** following extensive suppression of serum iPTH levels *via* pharmacological or surgical means for secondary hyperparathyroidism, there is a subsequent decline in osteoblast viability. This decline contributes to decreased bone turnover and the onset of ABD. Prolonged exposure to uremic toxins (PBUTs), diabetes, inflammation, CKD, malnutrition, and hyperphosphatemia worsen the condition of low bone turnover by diminishing PTH signaling. **②** for enhancing bone turnover and improving bone microarchitecture through increased osteoblast activity, the consideration of bone anabolic medications like teriparatide and abaloparatide is warranted. Addressing vitamin D deficiency with nutritional supplementation can restore osteoblast viability, while the use of AST-120 may prove beneficial in lowering levels of uremic toxins (PBUTs). Furthermore, the incorporation of anti-inflammatory/antioxidant agents may offer additional support in this regard. **③** In our meta-analysis, we utilized the Hierarchical Summary Receiver Operating Characteristic (HSROC) methodology to synthesize the diagnostic accuracy of a biomarker across various studies. The statistical analysis utilized hierarchical modeling methods to accommodate the diversity among studies. Analysis of positive likelihood ratio data indicates that an iPTH level below 150 pg/mL and a BALP level of 20 ug/L or less is indicative of the expected diagnosis of ABD. **④** We recommend that achieving an enhanced treatment response in ABD may involve consistent monitoring of serum bone biomarkers, with BALP levels maintained above 21 ug/L and iPTH levels not falling below 150 pg/mL. Furthermore, subsequent iPTH levels should ideally remain below 300 pg/mL.

Based on our meta-analysis findings, we recommend diagnosing ABD if iPTH is < 150 pg/mL and BALP is ≤ 20 μg/L, observed consistently over two to three consecutive blood tests within six months. Effective monitoring during pharmacological treatment entails regular assessments to ensure BALP levels remain above 21 μg/L and iPTH levels maintain a minimum of 150 pg/mL, which can enhance treatment response. Subsequent iPTH levels should ideally not exceed 300 pg/mL. Considering that current treatments for CKD-MBD may influence BALP and iPTH levels, further clinical exploration is warranted. Future guidelines should incorporate specific iPTH and BALP targets, while interventional studies should assess pharmacological agents’ impact on iPTH and BALP in CKD patients with ABD (Figure 3).

### VI-5. Other bone formation markers

Procollagen Type 1 N-terminal propeptide (P1NP), a bone formation marker, holds promise as it is not cleared by the kidneys [134]. Unfortunately, the biomarkers are not widely available for clinical use. Using a variety of biomarkers to predict bone turnover, research shows positive predictive values ranging from 50% to 90% for high and low turnover states which were verified by histomorphometry [79,80,135]. Osteoprotegerin (OPG), acting as a soluble decoy receptor, inhibits osteoclast activation, thereby reducing bone resorption. Elevated OPG levels are considered a contributor to skeletal resistance to PTH, leading to the low turnover state observed in ABD [136]. Bone morphogenetic proteins (BMPs) play a vital role in the communication between osteoblasts and osteoclasts, impacting bone remodeling. They stimulate osteoblast differentiation, mineralization, and viability, contributing to maintaining healthy bone [137]. Reduced BMP production and circulating levels are linked to slow bone turnover in ABD [136]. Leptin, an adipokine, actively regulates bone metabolism, influencing bone turnover. Peripheral effects of leptin are implicated in the reduced bone turnover observed in ABD [8,138]. In addition, N-terminal truncated PTH molecular species can counteract whole PTH effects on bone, potentially contributing to the low bone turnover in ABD [136].

### VI-6. Bone resorption markers

Bone resorption biomarkers are vital for detecting ABD, where bone becomes resistant to or experiences over-suppression of PTH. A reliable diagnosis of ABD requires measuring additional bone resorption biomarkers alongside aberrant PTH levels [139].

Our previous research established that serum tartrate-resistant acid phosphatase 5b (TRACP5b) serves as a clinically significant marker for tracking osteoclastic activity and bone resorption rates [140]. Derived from osteoclasts, TRACP5b serves as a valuable indicator, aiding in the identification and understanding of ABD [1,117]. α-Klotho, a co-receptor for phosphaturic FGF-23, aids in promoting phosphate excretion released by bones in response to elevated phosphate and 1,25(OH)_2_D levels. Independently of klotho, FGF-23 inhibits bone mineralization in osteoblasts by reducing the expression of tissue nonspecific ALP [141]. Reduced α-Klotho expression in CKD leads to FGF-23 resistance, contributing significantly to CKD-associated abnormalities [2]. On the other hand, elevated osteoprotegerin (OPG) levels contribute to skeletal PTH resistance in ABD. Acting as a decoy receptor, OPG binds to RANKL, inhibiting osteoclast action and reducing bone resorption [17].

### VI-7. Limitations of biomarkers in ADB of CKD

Biomarkers crucial for diagnosing CKD-related bone disorders like ABD and osteitis fibrosa cystica (OFC) face limitations due to overlapping patterns of low bone density [29]. In CKD, elevated PTH levels are common due to impaired calcium and phosphate regulation by the kidneys. However, some CKD patients exhibit high iPTH levels alongside low BALP levels, indicating potential PTH resistance, where bone tissue shows reduced responsiveness to PTH [116]. Detecting ABD depends on the disease stage, possibly causing diagnostic delays in early ABD with minimal changes [142]. The introduction of vitamin D analogs in 1980 reduced osteomalacia incidence in CKD patients [143,144]. Currently, ABD is the most prevalent CKD-MBD with low bone turnover.

## VII. Navigating treatment challenges

Current evidence challenges the assumption that low bone turnover in CKD consistently leads to negative outcomes. The link to osteoblast suppression remains unclear [59,75]. Treatments like calcimimetics, postparathyroidectomy follow-up, active vitamin D, and anti-resorptive agents show promise in achieving a more balanced reduction of bone turnover [145–148]. However, evidence lacks support for anti-resorptive improving fracture risk or mortality in CKD-MBD patients with ABD. Hesitancy among physicians to prescribe strong anti-resorptive in CKD-MBD stems from limited clinical trial evidence, emphasizing the need for cautious reevaluation of low bone formation in advanced CKD [149,150]. Differentiating ABD from age-related osteoporosis is crucial, advising against indiscriminate anti-resorptive use in CKD patients with low bone turnover [149,151,152].

Beyond support, the skeleton buffers acid provides a stem cell niche, and secretes vital hormones for metabolism. Medications altering bone physiology affect skeletal and bodily systems, with varied effects based on duration [153]. In osteoporosis, bisphosphonates reduce bone formation rates significantly, and long-term use leads to crack accumulation, impacting strength in biopsies [154–156]. Varied density in microscopic bone regions challenges the assumption that higher BMD always translates to increased strength. Directly addressing iPTH levels may be more appropriate, as demonstrated in a *post hoc* analysis of a cinacalcet trial. This analysis indicates a significant decrease in fracture rates in dialysis patients with high iPTH [157]. These findings emphasize the significance of taking into account both the quality and quantity of bone in cases of both low and high bone turnover [158].

It is unclear whether anti-resorptive drugs are effective in patients with osteoporosis and low bone formation. Inhibiting low bone resorption may increase mineral density, but bisphosphonates may not enhance strength without remodeling. Alendronate showed no fracture rate benefit in lower bone formation tertiles but decreased fractures in the highest turnover tertile [159]. Bisphosphonates minimally impacted bone density in CKD stages 4 and 5 [160]. Prolonged Denosumab use led to uniform bone mineralization and increased hip density over 10 years, but discontinuation resulted in a 12.7% decrease, causing vertebral fractures [161,162]. Anabolic treatment is recommended for advanced CKD patients with ABD [5].

### VII-1. Anti-osteoporosis treatment in the context of low bone turnover (LBT)

Recent nephrology consensus on osteoporosis in advanced CKD suggests the potential reduced efficacy of anti-resorptive therapy in LBT patients. However, positive responses from diabetics and Denosumab’s prolonged impact challenge this notion [5,31]. Anabolic agents are proposed for CKD with LBT, offering faster fracture risk reduction [163,164]. PTH analogs in LBT show positive effects, though dosing uncertainties persist [165]. Romosozumab, a sclerostin antagonist, inhibits resorption and promotes osteoblast-driven bone formation with potential cardiovascular considerations [166,167]. Personalized therapy, considering medical history, is crucial, especially in off-label use, requiring thorough patient education and documentation [168].

## VIII. Current management strategies for ABD

Anti-resorptive benefits for the high-turnover bone disorder but raise concerns in CKD with ABD, especially in severe hypoparathyroidism [169]. No studies directly address their effects on CKD patients with ABD. Kidney-eliminated bisphosphonates may persist, advocating for lower doses over shorter durations. CKD patients are more prone to atypical femur fractures with anti-resorptive use. Precision in therapy selection, guided by bone turnover markers, is emphasized for a safe and effective approach [5].

### VIII-1. Potential therapeutic considerations of ABD in CKD

Optimal measures to prevent ABD involve medications enhancing bone mass, cortical bone size, trabecular structure, connectivity, and material properties without hindering micro-damage repair [121]. In CKD, accumulated uremic toxins compromise bone quality, requiring a reduction in toxin levels to minimize fractures. AST-120, an oral adsorbent, is clinically employed to reduce serum IS levels and decelerate renal function decline. This helps mitigate the deleterious effects on cortical bone in CKD [170]. Our recent clinical investigation demonstrates that AST-120 effectively alleviates uremic pruritus by lowering serum indoxyl sulfate levels and inflammatory cytokines among hemodialysis patients. Upon analyzing the follow-up data, we observed a correlation between the reduction in serum indoxyl sulfate levels and a decrease in serum iPTH levels. This suggests a potential indirect effect of indoxyl sulfate on PTH hypo-responsiveness, which could contribute to the development of adaptive hyperparathyroidism in chronic kidney disease (CKD) [171]. Other pharmacological therapies for ABD include anabolic agents, parathyroid hormone analogs, and nutritional approaches like reducing serum phosphorus levels and using nutritional vitamin D [2,172].

### VIII-2. Correcting vitamin D deficiency

Calcitriol plays a significant role in regulating osteoid mineralization, prompting the clinical use of drugs targeting the PTH/vitamin D axis to prevent bone abnormalities in CKD. However, studies on vitamin D’s effects on histologic mineralization in dialysis patients yield conflicting results [173,174]. In human osteoblasts, 1-α-hydroxylase is vital for differentiation, enhancing 1,25(OH)_2_D_3_ production and promoting osteopontin, osteocalcin, and alkaline phosphatase expression [175]. Additionally, short-term 1,25(OH)_2_D_3_ administration enhances Wnt signaling, promoting osteoblast viability and differentiation [176]. In osteoblasts, the 1,25(OH)_2_D_3_–VDR (vitamin D receptor) effective binding influences osteogenesis through canonical Wnt signaling, promoting RunX2 and increasing osteocalcin expression [177,178]. Furthermore, it stimulates the expression of RunX2, subsequently increasing the levels of osteocalcin and osteopontin [179]. During bone remodeling, 1,25(OH)_2_D_3_ stimulates osteoclast differentiation by inducing RANKL and M-CSF expression [179].

Our previous study demonstrated that calcitriol treatment increased serum Wnt 10b and P1NP levels while reducing Trap 5b levels in hemodialysis patients. In cell culture studies, calcitriol exhibited a dose-dependent reduction in osteoclast differentiation and promoted the release of Wnt 10b from suppressed osteoclasts. These findings suggest that short-term, high-dose calcitriol, used in the treatment of secondary hyperparathyroidism (SHPT), may enhance bone formation by inhibiting osteoclast activity and stimulating osteoblast function through elevated Wnt 10b levels [147]. Calcitriol, while modestly promoting osteoclast maturation, strongly inhibits osteoclast lineage commitment from its progenitor monocytes [180]. Long-term exposure to active vitamin D compounds suppresses RANKL expression in osteoblastic cells, potentially decreasing RANKL activity and osteoblastic cellularity with daily administration [181]. Moreover, active vitamin D efficiently suppresses PTH, making it contraindicated in CKD patients with low bone turnover, as it could exacerbate low PTH levels and worsen bone turnover further.

However, vitamin D obtained through nutrition (native vitamin D) activates osteoblasts and helps maintain serum 25(OH)D levels, supporting its use in patients with osteoporosis [158]. In long-term hemodialysis patients with low bone turnover, the initial bone histology revealed trabecular surfaces covered with scanty osteoid but lacking active osteoblasts. Following 86 weeks of calcifediol treatment, sporadic regions displayed signs of active remodeling, characterized by the presence of accumulated osteoid lamellae and some areas of mineralized bone [182]. These results demonstrate that calcifediol partially restores osteoblast viability in chronic kidney disease with ABD. Hence, excessive active vitamin D treatment for secondary hyperparathyroidism (SHPT) should be avoided. Instead, supplementation with native vitamin D to prevent deficiencies may improve bone health [8,121,183].

### VIII-3. Anti-resorptive medications

Anti-resorptive drugs, such as bisphosphonates, benefit high bone turnover disorders by reducing bone turnover. While bisphosphonates show therapeutic benefits in CKD stages 1-3b and post-transplantation, limited data for stages 4-5D focuses on hypercalcemia treatment [184–187]. In advanced CKD, risks involve accelerated GFR decline and the potential exacerbation of low turnover states [188]. Monitoring bone turnover markers is crucial, as PTH post-treatment may rise, proving unreliable for indicating bone turnover [189]. Caution is advised in using bisphosphonates for CKD stages 4-5 with declining eGFR, despite potential benefits for the high bone turnover disorder [190].

Denosumab shows greater bone density gains postbisphosphonate treatment, even with lower turnover at bisphosphonate commencement [100,191]. The impact of baseline turnover on denosumab efficacy remains uncertain, with recent studies using an iPTH upper limit of 240 pg/ml for analysis [192]. In a recent trial, 1-year therapy with alendronate or denosumab had no vascular impact in dialysis patients [193]. Denosumab, especially, may lead to hypocalcemia, more likely with elevated baseline turnover but manageable with calcium or vitamin D supplementation [194,195]. Denosumab’s rapid offset requires continuous administration or alternative therapy to prevent swift bone loss and increased fracture rates [196]. Monitoring bone turnover markers aids in tracking denosumab’s effects [197].

Raloxifene, a selective estrogen receptor modulator used for osteoporosis, demonstrates anti-resorptive properties and boosts osteoblast activity [198]. A three-year study in advanced CKD demonstrated an increase in BMD and a decrease in the risk of vertebral fractures, particularly in cases of mild to moderate CKD [199,200]. Patients with a history of thromboembolic events should exercise caution, even though the thrombotic risk is lower compared to estrogen therapy [201].

### VIII-4. Anabolic medications

Anabolic agents like teriparatide and abaloparatide enhance bone formation, reducing fracture risk in CKD patients [29,202]. Teriparatide, a synthetic parathyroid hormone, stimulates osteoblast activity, increasing BMD and mitigating fractures in either postmenopausal or glucocorticoid-induced osteoporosis [29,203–205]. However, CKD use requires caution due to potential hypercalcemia and hyperphosphatemia [165,206,207]. Abaloparatide, a synthetic PTH analog, is effective in reducing the risk of fractures and enhancing BMD in postmenopausal osteoporosis [208]. While these agents improve bone density, especially in mild to moderate CKD, their use in CKD-associated ABD lacks thorough study, requiring further research to assess potential benefits and risks [204,209].

Romosozumab, a monoclonal antibody targeting sclerostin, promotes bone health by blocking its inhibitory effects on formation, increasing bone density and strength [210]. Clinical trials confirm efficacy in postmenopausal osteoporosis, providing dual anabolic and anti-resorptive effects [211]. In early CKD, elevated sclerostin may impact vasculature and bones, potentially preventing vascular calcification [212]. While observational studies in dialysis patients show benefits, caution is needed due to safety signals in osteoporosis patients related to cardiovascular events [213–216]. Observational studies in Japanese dialysis patients show elevated BMD and fewer cardiovascular events with romosozumab [217]. However, broader clinical utilization is impeded by apprehensions regarding cardiovascular events and potential carcinogenic effects [217–220]. Further controlled studies are imperative to provide a conclusive evaluation.

### VIII-5. Prospective beneficial of combination therapy

For the complex conditions of CKD-MBD and ABD, a balanced approach combining anabolic and osteoporosis treatments, including anti-resorptive agents, is recommended to enhance bone quality and reduce fracture risks in some CKD patients [221–223]. Personalized therapy decisions should consider the patient’s clinical condition, CKD stage, and fracture risk.

## IX. Therapeutic monitoring

Guided by bone turnover markers, precision in anabolic therapy is crucial for safety and efficacy. BALP, reflecting bone formation, aids in assessing ABD treatment effects [2,8]. While not directly linked to ABD, intact PTH is a vital CKD-MBD biomarker, offering insights into overall bone metabolism. Monitoring iPTH levels contributes to a comprehensive ABD treatment evaluation [16]. Recognition of proposed levels involves maintaining BALP above 21 ug/L and iPTH levels not falling below 150 pg/mL during treatment (ClinicalTrials.gov Identifier: NCT04522622). The duration of therapy, be it anabolic agents or others, remains uncertain and should be individualized. Regular DXA monitoring every 2 years provides valuable insights and discontinuation of anabolic or anti-resorptive therapy may be considered with significant improvements in BMD or histomorphometry [223].

## X. Conclusions

In CKD patients with ABD, underlying conditions like malnutrition, diabetes, and CKD contribute to oxidative stress, hyperphosphatemia, deficiencies in calcitriol and magnesium, inflammation, and the retention of protein-bound uremic toxins (PBUTs). These possible correctable factors may induce PTH hypo-responsiveness, resulting in reduced PTH signaling low bone turnover and a negative bone mineral balance. Conversely, treatments such as calcium overload, active vitamin D analogs, and calcimimetics may over-suppress PTH, also leading to low bone turnover [224,225]. Additionally, anti-resorptive medications decrease bone turnover, leading to brittle bones despite a positive mineral balance, impairing bone quality [169]. Therapies aim to increase bone turnover and achieve a positive mineral balance by managing disease-related factors directly or avoiding PTH over-suppression. Both the factors leading to suppressed bone turnover and the inherent low bone turnover itself could contribute to the observed association with outcomes [3]. Anabolic therapy has been proposed, from a pathophysiological perspective, as a means to enhance turnover and increase bone mass in individuals with advanced CKD and low bone turnover. Nevertheless, there remains uncertainty regarding its safety profile, necessitating further investigation [224].

Precision medicine utilizes biomarkers to assess CKD-related ABD severity, monitor treatment response, and predict outcomes. It enables personalized plans, incorporating lifestyle changes, medications, anabolic agents, and nutritional vitamin D. Stratifying by risk allows proactive interventions, preventing ABD progression. Bone turnover markers, like BALP and iPTH, crucial for diagnosis and treatment, reveal insights into bone formation and resorption rates. These biomarkers guide tailored interventions, optimizing treatment strategies effectively for addressing mineral and bone disorders in CKD-related ABD.
